# Barriers, facilitators and attitudes influencing health promotion activities in general practice: an explorative pilot study

**DOI:** 10.1186/1471-2296-14-20

**Published:** 2013-02-09

**Authors:** Wytske W Geense, Irene M van de Glind, Tommy LS Visscher, Theo van Achterberg

**Affiliations:** 1 Scientific Institute for Quality of Healthcare, IQ healthcare, Radboud University Nijmegen Medical Centre, the Netherlands, P.O. Box 9101, Nijmegen, HB, 6500, The Netherlands; 2 Research Centre for the prevention of overweight, Zwolle, Windesheim and VU University, Zwolle/ Amsterdam, The Netherlands

**Keywords:** Life styles, Family practice, Family nursing, Implementation, Qualitative study, Attitude

## Abstract

**Background:**

The number of chronically ill patients increases every year. This is partly due to an unhealthy lifestyle. However, the frequency and quality of (evidence-based) health promotion activities conducted by Dutch general practitioners (GPs) and practice nurses (PNs) are limited. The aim of this pilot study was to explore which lifestyle interventions Dutch GPs and PNs carry out in primary care, which barriers and facilitators can be identified and what main topics are with respect to attitudes towards health promoting activities. These topic areas will be identified for a future, larger scale study.

**Method:**

This qualitative study consisted of 25 semi-structured interviews with sixteen GPs and nine PNs. ATLAS.ti was used to analyse the transcripts of the interviews.

**Results:**

All GPs and PNs said they discuss lifestyle with their patients. Next to this, GPs and PNs counsel patients, and/or refer them to other disciplines. Only few said they refer patients to specific lifestyle programs or interventions in their own practice or in the neighbourhood. Several barriers and facilitators were identified. The main topics as barriers are: a lack of patients’ motivation to make lifestyle changes, insufficient reimbursement, a lack of proven effectiveness of interventions and a lack of overview of health promoting programs in their neighbourhood. The most cited facilitators are availability of a PN, collaboration with other disciplines and availability of interventions in their own practice. With respect to attitudes, six different types of GPs were identified reflecting the main topics that relate to attitudes, varying from ‘ignorer’ to ‘nurturer’. The topics relating to PNs attitudes towards health promotion activities, were almost unanimously positive.

**Conclusion:**

GPs and PNs all say they discuss lifestyle issues with their patients, but the health promotion activities that are organized in their practice vary. Main topics that hinder or facilitate implementation are identified, including those that relate to attitudes of GPs and PNs.

## Background

Chronic noncommunicable diseases, like diabetes, cardiovascular diseases and chronic lung diseases, are the leading cause of death and disabilities worldwide [1]. Besides aging of the population, an unhealthy lifestyle plays an important role in the increasing prevalence of chronic diseases. Health promotion and prevention, such as the discouragement of smoking, alcohol abuse and obesity are necessary to reduce the personal and societal consequences [2]. Several studies have shown that general practitioners (GPs) are, among others, capable to fulfil these tasks because they have relatively many patient contacts, they are perceived to be the most reliable formal source of information and can identify patients who are at risk at an early stage [3-5]. In recent decades practice nurses (PNs) have taken an increasing role in general practice as well as in providing lifestyle counselling to certain patients groups like diabetics. Health promotion activities and lifestyle advice given by GPs and PNs [6-9] can lead to a decrease in alcohol use [5], smoking cessation [3,10], increased physical activity [11,12] and weight reduction [13]. Few evidence-based health promotion activities are carried out in Dutch general practice [14-18], thus indicating the need to look into barriers and facilitators in this field of healthcare practice.

Various quantitative studies have been conducted into barriers GPs experience and their attitudes pertaining to health promotion activities in general practice [6,11,19-21]. However, qualitative studies, leading to more understanding of relevant factors and covering both subjects are limited [8,22-26] or conducted abroad [8,22,25,27,28].

Our pilot study aimed to explore which health promotion activities Dutch GPs and PNs carry out in primary care, which barriers and facilitators can be identified and what main topics are with respect to attitudes towards health promotion activities. These topic areas will be identified for a future, larger scale study.

## Methods

### Recruitment

Five health regions [29] in the Netherlands were selected from which GPs and PNs were invited to be interviewed. The selection of these regions was based on regional spread of health promotion research institutes and on contact through related previous research performed by Van de Glind and Heinen et al. (2012) [30]. Purposive sampling was used to include at least three GPs (preferably one in a solo practice, one in an healthcare centre and one in a general practice) and one PN (in a general practice or health care centre) in every region. Firstly we sent emails to professors of general practice medicine and to umbrella GP organizations to obtain names of relevant GPs. In case of limited or no response, the second strategy was to approach PNs in general practices and ask them if they wanted to participate and if they knew relevant GPs. Additionally, letters were sent to GPs and PNs and after one week reminders were conducted by phone. Anonymity and confidentiality was ensured. Verbal informed consent was obtained.

The Central Committee on Research Involving Human Subjects (CCMO), the Nationwide committee supervising al regional committees in the Netherlands, provides a very clear instruction on when to present research plans to medical ethics committees. It is encouraged that researchers check these instructions first. In our case, the national instructions very clearly indicate how this was not a study subject to the Medical Research Involving Human Subjects Act (WMO).

### Interviews

Semi-structured interviews were conducted by WG in March and April 2011 in the GPs’ and PNs’ consulting rooms and took approximately 30 minutes. The topic list was developed using the related literature and the experiences of the research team. Three subjects were discussed in the interviews.

1. Health promotion activities in general practice

•Activities used in general practice pertaining to a healthy diet, physical activity, losing weight, stop smoking and reducing alcohol use

•Origin of activities

•Since when are activities carried out

•Patient groups

•Related costs

•Changes in activities

•Results / effects of activities

2. Barriers and facilitators

•Barriers pertaining to the delivering of health promotion activities

•Facilitators pertaining to the delivering of activities

•Requirements/ fittings for the future

3. Attitude of the GP or PN

•Attitude of GP/ PN pertaining to their role in health promotion

•Knowledge and skills pertaining to carrying out health promotion activities

•Attitude of GP/ PN pertaining to the patient’s responsibility

•Balance between promotion of health and interfering in someone’s life

Firstly, using structured questions, the GPs and PNs were separately asked what they do to reduce the use of alcohol, smoking cessation, to promote a healthy diet, physical activity and losing weight. The selection of these lifestyle subjects was based on the priorities of Dutch public health policy [31].

Secondly, open questions were used to ask GPs and PNS about the barriers and facilitators they experienced. Thirdly, their attitudes towards their role, pertaining to health promotion activities in general practice, were asked using open-ended questions.

All interviews were audio-taped and transcribed verbatim by WG. The first five interviews were both read by WG and IvdG in order to refine questions. The last question about the balance between promotion of health and inferring in someone’s life was added after the first five interviews.

### Data analysis

The transcripts of the interviews were coded by WG using ATLAS.ti 6x. To increase validity and to reach consensus, the first seven transcripts were also coded by IG. At the end, IvdG checked all the codes and discussed them with WG.

Characteristics and the health promotion activities in general practice were analysed using fixed codes based on the topic list.

Influencing factors were categorised using open coding. Codes were connected to themes and subthemes. An example of a theme is the requirements of health care insurance companies that GPs have to meet to receive reimbursements. The subthemes within this theme were for example the time consuming registration, and accredited courses PNs have to attend. (Sub)themes were subdivided into barriers and facilitators regarding the delivering of health promoting activities. Only barriers and facilitators mentioned by more than two participants were reported in this study. To further structure the identified barriers and facilitators, five different categories were used (category of patients, general practice, attitude of the GP and PN, health promotion programs and health care system) and these were discussed in the research team again.

Attitudes were categorized using open coding as well. Related codes were connected to (sub)themes. In the first way, codes of attitudes were divided into hindering and facilitating attitudes. However, it was not possible to subdivide all the attitude codes into barriers or facilitators; it did not yield satisfying results. Therefore, transcripts of GPs and PNs were read again to find patterns, similarities and differences in their attitudes that corresponded to the codes and themes. Then, a pattern of six types of attitudes of GPs was identified. This study adheres to the RATS guidelines on qualitative research [32].

## Results

In total, we interviewed sixteen GPs and nine PNs of seventeen different practices. In four different regions we interviewed three GPs of different practices and one PN. In one region we interviewed four GPs and five PNs. The characteristics are illustrated in Table 1.

**Table 1:** Characteristics of included GPs and PNs

**Practice (N=17)**	General practice (N=8)*	
	Health care centre (N=8)**	
	Therapeutic centre (N=1)***	
Function (N=25)	GP (N=16)	PN (N=9)
Gender (N=25)	GP Female (N=8)	PN Female (N=9)
	GP Male (N=8)	
Working status (N=25)	GP Part-time (N=6)	PN Part-time (N=8)
	GP Fulltime (N=10)	PN Fulltime (N=1)

*Presence of GPs and PNs.

**Presence of GPs, PNs and several disciplines such as physiotherapists, dieticians, psychologists and social workers.

***Anthroposofic health centre: presence of (Anthroposofic) dietician and physiotherapist.

The majority of the GPs and minority of the PNs worked fulltime and two thirds of the participants are women. Seventy-five GPs did not want to participate in this study due to a lack of time (N=68), lack of interest (N= 6) or pregnancy (N=1).

### Health promotion activities in general practice

Table 2 shows that all GPs said that they actively ask patients with lifestyle related symptoms about their lifestyles. Most GPs said they give patients lifestyle advice as well. The advise includes information about the consequences of their unhealthy lifestyle and stimulates awareness of the problem, motivation aspects and what healthy behaviour consists of. Moreover, the GP or PN try to find out why a patient has an unhealthy lifestyle.

**Table 2:** Most often mentioned activities to promote a healthy lifestyle and of related patient groups

	**Actions among GPs an PNs (N=17)**	**Related patient groups**
**Alcohol reduction**	Give advice (N= 17)	Alcoholics (N= 16)
	Give a referral to addiction treatment (N=15)	Diabetics (N=10)
	Individual lifestyle counselling by GP (N=4)	Cardiovascular patients (N=9)
**Stop smoking**	Give advice (N=17)	COPD patients (N=14)
	Individual lifestyle counselling by PN (N=15)	Diabetics (N=13)
	Prescribe medication (N=13)	Cardiovascular N=12)
**Promotion exercise**	Give advice (N=14)	COPD patients (N=12)
	Give a referral to exercise program physiotherapist (N=12)	Diabetics (N=11)
	Refer to physiotherapist (individual) (N=10)	Cardiovascular patients (N=10)
**Promotion healthy diet**	Give advice (N=16)	Overweight/ obese patients (N=15)
	Give a referral to dietician (N=15)	Cardiovascular patients (N=12)
	Individual lifestyle counselling by PN (N=13)	Diabetics (N=10)
**Losing weight**	Give advice (N=16)	Overweight/ obese patients (N=17)
	Give a referral to dietician and physiotherapist (N=13)	Diabetics (N=4)
	Give a referral to surgeon (N=2)	

As one PN stated: *“I discus what is healthy or accepted, and If a patient drinks too much I try to find out the underlying reason”.*

If patients are motivated to change their behaviour, GPs and/or PNs give advice about the options how to change their unhealthy behaviour and try to help them. Most of the PNs said they use motivational interviewing. The information they provide is based on the national general practitioner guidelines.

Besides this, GPs or PNs said they refer patients to other disciplines, for instance, to the dietician, physiotherapist, psychologist or addiction care. As one GP expressed:

"‘I have to say, I give a referral quite easily to Iriszorg (addiction care). In the past I tried to counsel alcoholics myself, but it was very disappointing’."

Some of the GPs mentioned giving patients referrals to nationally disseminated health promotion programs. Others said they had referred patients to these programs in the past, but due to lack of proven effectiveness and reimbursements they stopped referring. Moreover, GPs expressed they prefer to offer health promotion programs in their own practice, especially exercise programs in collaboration with physiotherapists, instead of national programs outside their practice. They stated it is easier accessible and more familiar for patients.

The activities of the PNs are listed in Table 2 as well. PNs reported they give lifestyle counselling to chronically ill patients in general practice, mainly to patients with diabetes, COPD and cardiovascular diseases. PNs actively ask these patients about their lifestyles and give them lifestyle advice. Furthermore, PNs said they give lifestyle counselling for patients who want to quit smoking.

### Barriers and facilitators experienced by GPs and PNs

During the interviews, GPs and PNs mentioned 41 different barriers with respect to delivering health promotion activities in general practice (see Table 3).

**Table 3:** Barriers and facilitators for delivering health promotion activities

	**PATIENT**	**GENERAL PRACTITIONER/ GENERAL PRACTICE**	**ATTITUDE GENENERAL PRACTITIONER**	**HEALTH PROMOTION PROGRAM**	**HEALTHCARE SYSTEM/ GOVERNMENT**
**BARRIERS**	Lack of patients’ motivation to change unhealthy behaviour *	Results are difficult to measure	Patients do not appreciate it when GPs of PNs discuss their lifestyles	Lack of proven effectiveness of health promotion programs	The hours of PN are not fully compensated financially
	Unhealthy lifestyle is socially accepted, especially drinking alcohol	Lack of skills among GPs and PNs to discuss lifestyle and develop health promotion programs	Group sessions seems to be more effective compared with individual counselling, but most of the health promotion programs in general practice are individual	Lack of overview of health promotion programs	Lack of reimbursements and subsidies to start new health promotion programs in general practice
	Patients deny or lie about their actual lifestyles	Lack of time among GPs to discuss lifestyle with patients and develop health promotion programs	GPs state discussing lifestyles is a waste of time	Lack of continuity of health promotion programs, due to short-term reimbursements and subsidies	GPs have to meet too many strict requirements of healthcare insurance companies, to receive reimbursement and subsidies (e.g. registration, accredited courses)
	Patients are unaware of their unhealthy lifestyles	Dietician and addiction care consultant disappear due to lack of patients	Consultation hours are more focused on treatment instead of on prevention	Not all patients can be reached in general practice	Lack of trust among GPs and PN in reimbursement and subsidies due to continuous changes
	Patients experience barriers to live a healthy lifestyle (e.g. co-morbidity, lack of time)	GPs do not give patients referrals and motivate their patients as much as they can	GPs are sceptical about the effects and results of discussing lifestyle	Programs are not accessible, due to narrow inclusion criteria and affordability of programs	Contradictory policy of Dutch government (e.g. expensive healthy food, inconsistent smoking policy)
	Behavioural change is a complex process for patients, especially when the environment does not change	Due to unhealthy behaviour of GPs and PNs (especially alcohol use) it is difficult to discuss lifestyles with patients	GPs think lifestyle is not important	Lack of health promotion programs	GPs and patients have to find out reimbursement and subsidies from insurance companies themselves
	Letting patients pay contribution for health promotion programs does not work, especially not among low SES patients	Motivation of GPs and PNs decrease due to disappointing results		Programs are not accessible for patients due to waiting lists	Lack of collaboration between hospital and general practices with regard to health promotion activities
	Due to stigma patients are not going to addiction care	Lack of collaboration between disciplines			Health promotion activities in general practice are not rewarded
	Patients do not go to health promotion programs due to geographical barriers (E.g. distance to program)	Lack of room and housing			Contradictory information from insurance company towards patients
		GPs forget to ask about lifestyles			
**FACILITATORS**	Patients who are aware of their own lifestyles and who are motivated to change their lifestyles is a motivation for GPs and PNs	Availability of PNs in general practice: he/she has more time than GPs and plays a central role	GPs thinks it is worthwhile to discuss lifestyle with patients	Health promotion programs in general practice are familiar for patients	Reimbursements and subsidies determine participation and development of health promotion programs
	Let patients do what they want to do; there is a bigger chance they will succeed	More collaboration and feedback due to availability of physiotherapist and dietician in general practice	GPs state it is part of their job to promote a healthy lifestyle	Easy accessible health promotion programs due to broad inclusion criteria and affordability	Umbrella of GP organization develop health promotion programs and clear policy
	Patients are more motivated when they have insight in their results (e.g. blood sugar level)	Sufficient staff for developing and conducting lifestyle programs	GPs and PNs think they are skilled to discuss lifestyle with patients	Continuity of health promotion programs	
	Patients are more motivated to participate in a lifestyle program when they have to pay contribution	Familiarity between patients and GP and PNs is an advantage to discuss lifestyle	Healthy lifestyle of GP and PNs is a role model for patient	Best way to discuss lifestyle is in an open manner, not by using a protocol	
		Sufficient room and accommodation		Proven effectiveness of health promotion programs	
		Enthusiastic colleagues to develop and deliver lifestyle programs		Overview/ social map of disciplines and health promotion programs	
		Structured registration and labelling of patients at risk provide an overview for GPs		Availability and collaboration with sport facilities	

*Most cited factors are at the head of the table. E.g. ‘Lack of motivation by patients’ is mentioned by nineteen participants.

Firstly, GPs and PNs mentioned barriers regarding their patients: patients were not motivated to change their unhealthy lifestyles and deny and lie about their behaviour. Furthermore, some patients were said to not appreciate discussing their lifestyles and GPs and PNs stated that an unhealthy lifestyle is socially accepted, especially drinking alcohol.

Secondly, GPs and PNs experienced barriers related to their own practice: they stated they have a lack of time in their consultations to discuss lifestyle issues with their patients. Moreover, they mentioned there is a lack of corporation with other disciplines.

Thirdly, GPs and PNs stated they experience problems regarding the content of health promotion programs. According to them, there is a lack of proven (long-term) effectiveness, and next to this, there is no overview of existing programs in the neighbourhood.

Fourthly, respondents stated that reimbursement of the programs is a barrier: a lot of health promotion programs are not financed or only financed for a short period. Due to that, there is lack of continuity, there are no long- term programs and therefore there is uncertainty and lack of trust to implement new initiatives.

As one GP said:

"‘This is the second year that we offer this program to our patients. Now, the program is granted…as long as it lasts’."

Moreover, programs are not always accessible for patients with a low socio-economic status (SES) and if a general practice receives a reimbursement, they have to meet strict and time-consuming requirements from insurance companies or research institutes. Examples are the registration of performed activities, the inclusion of patients and the required education and skills of practice nurses to take certified courses and training.

At last, contradictory policy of the government is an experienced barrier as well: for instance GPs mentioned the inconsistent smoking policy (in 2008 smoking was banned in all restaurants, clubs and hotels but this was overturned in 2012).

As one GP expressed:

"‘We are glad, because we get support and reimbursements to motivate people to stop smoking. However, when I heard on the radio “It is allowed to smoke in small cafes again”, I thought; “What do they want?!”’"

Most cited facilitators are the availability of PNs in general practices, reimbursement of lifestyle programs, having programs in their own practice, the development of programs by umbrella GP organizations and collaboration with several disciplines in general practice, like dieticians, physiotherapist and psychologist (see Table 3).

Remarkably, there are some contradictions in the mentioned barriers and facilitators. With respect to the contribution patients have to pay for certain health promotion programs, some GPs and PNs stated this is a barrier because programs are not accessible for everyone, especially not for patients with a low SES. However, others think it is a facilitator, because if patients have to pay for a program they will be more motivated to change their behaviour. Another contradiction is whether GPs and PNs own healthy behaviour will influence health promotion activities positively or not. It could be a barrier, especially with regard to drinking alcohol, as one GP expressed:

"‘We do very little about reducing alcohol intake by patients, but it might have something to do with the fact that the doctor drinks alcohol as well’"

But it could also be a facilitator:

"‘I was a smoker. I have stopped. If I can do it, you can do it too’."

### Attitude of GPs and PNs

Although the majority of the GPs stated they are capable to fulfil the health promotion tasks, there are differences in their attitudes about their perceived roles and responsibilities. To describe and visualize these differences, six different types of GPs were identified: the ‘ignorer’, ‘adviser’, ‘confirmer’, ‘evangelist’, ‘interferer’ and ‘nurturer’ (see Table 4).

**Table 4:** Attitude of GPs toward discussing lifestyle with patients: a typology of GPs

	**IGNORER**	**ADVISER**	**CONFIRMER**	**EVANGELIST**	**INTERFERER**	**NURTURER**
**VISION**	Limited role of GP pertaining to the promotion of a healthy lifestyle. It is the task of the government to promote a healthy lifestyle. Reimbursement is a strong motivational factor for the delivering of intervention in general practice.	Health promotion and prevention are part of the job of a GP. Although GPs state their consulting hours are more focused on treatment instead of prevention, they think it is worthwhile to spend time on health education and counselling. But in the end patients make their own choices.	Self management among patients is very important in general practice. The PN has a central role to educate and counsel patients. The GP confirms the importance of healthy lifestyle and supports progress of behaviour change of patients.	The general practice carries out a lot of lifestyle programs. However the GP is sometimes sceptical about the effects. Nevertheless, they are sure that they can help at least some patients.	GP discusses lifestyle even if the patient has no related symptoms. They confront their patients with their unhealthy lifestyle. These GPs have a lot of lifestyle programs in general practice.	The role of the GP is like the role of a teacher/ educator or nurturer. GP’s have to educate their patients.
				The GP imposes standards of a healthy lifestyle also to his/her own life, to set a good example.		
**QUOTES**	*‘Those lifestyle interventions are not that important, in my opinion’.*	*‘Yes, eventually the patient is responsible; however as a GP I can provide patients with information’.*	*‘The plans for behaviour change are made between patient and PN (…). Our role is just to support and consolidate this. Even if they come with a cold, I say ‘You want to lose some weight. Well done!’*	*‘You want to create an atmosphere in which you radiate that and eventually it works somehow. Even if it is for later generations’.*	*‘If you see cigarettes in the patients’ breast pocket, do you have to say something about it or not? (.) If I am well acquainted with the patient, I do’.*	*‘You have to raise your patients and teach them how to deal with health in their life’.*
	*‘I can improve very little at the individual level’.*					
						*‘Patients are doing a lot of simple primitive things wrong (…) I may discuss that with my patients’.*
		*‘I think I am a health adviser’.*				
				*‘You must go on. On the one hand because there are (minimal) results and on the other hand because it is professional motivation. It is like ‘Médecins Sans Frontières’ an (international humanitarian organization); they go to a warzone, yes… and later on there is a new front. Shouldn’t they have gone out and help over there? You have to do something. You will help at least some individuals’.*		
					*‘I put all of them (fat ladies, WG.) on a weighing scale and I say: What do you think of it? (…) It’s a wakeup call. They don’t like it, but it has to be done, right?’*	
						‘*I think it makes sense to tackle bad lifestyle habits in order to prevent. And that we can say: well if you are not stupid, you have to get smart’.*
	*‘I would never discuss the importance of exercise etcetera if there is no policy on a national or regional level’.*	*‘Advisory, not mandatory. I give advice and patients can do with it what they want. It is their responsibility’.*				
			‘*We try to motivate patients, using the power of repetition and the fact we are a team’.*			
					*‘Sometimes I lay my hand on one of those pot bellies and say: When is the baby coming?’*	
	*‘I will change my working method if the insurance company offers me a reimbursement’.*					

The ‘ignorers’ stated that their role as a GP in health promotion activities, is limited and that the government should take more responsibilities. The ‘advisers’ mentioned that health promotion is part of their job and think it is worthwhile to spend time on giving lifestyle advices. The ‘confirmers’ emphasized the importance of the PNs and as a GP they confirm and support the plans made between the PN and patients. The ‘evangelists’ stated that even though they are sceptical about the effects, they are sure that they can help at least some patients. The ‘interferers’ discussed lifestyle with their patients by confronting them, even if there are no lifestyle related symptoms. The ‘nurturer’ stated that the GPs’ role is like the role of a teacher, to raise and educate their patients. In most cases a GP fits into one role. However, sometimes a GP fits into several roles, depending on the lifestyle factor she/he has to deal with. For example, a nurturer can become ignorer when there is a patient with alcohol problems. It may depend on the lifestyle topic, the problem, the patient and/ or the situation in which role a GP fits.

The PNs were also asked about their attitudes. They were unanimous. PNs stated that patients are always responsible for their own lifestyle and they quit giving support if a patient does not want to change or does not appreciate it when his or her lifestyle is discussed. The patient can come back when he or she is ready for it. Despite the fact they cannot help all patients and the results are minimal, PNs think discussing lifestyle is worthwhile. As one PN expressed:

"‘Lifestyle is more important than all the other things I do’."

## Discussion

The aim of this pilot study was to explore which lifestyle interventions Dutch GPs and PNs carry out in primary care, which barriers and facilitators can be identified and what main topic are with respect to attitudes towards health promotion activities.

GPs and PNs said that they advice and counsel their patients or refer them to other disciplines. They said that they perceive health promotion activities as part of their job. However, there were several topic areas identified as barriers to deliver health promotion activities. These barriers are related to the patient, the GP and the practice, attitudes, health promotion programs and the healthcare system. Six different and provisional types of GPs were identified, reflecting the main topics that are related to six different attitudes to health promotion activities. Topics brought up by PNs that are related to attitudes were practically unanimous and positive.

The main topic areas as barriers in this study are consistent with results of other studies. Most cited barriers mentioned in the literature are: lack of time [11,28,33], lack of confidence in providing advice and effectiveness of interventions [6,22,33], lack of reimbursements [11,19,28] and lack of patient compliance or motivation [11,21,22]. The lack of skills to discuss lifestyle is a major experienced barrier in the literature [28,34]. Participants in our pilot study only reported a lack of discussion skills regarding alcohol intake and specific diets. Another interesting finding is the fact that Lambe [28] recommends a national program in his study, due to the inequity of access. However, GPs and PNs in this study state the opposite; they prefer programs in their own practice instead of national programs outside their practice. The themes regarding attitudes that we identified in this study correspond with the existing literature. A study by Lambe [28] concluded that GPs think their practice is an ideal setting to deal with lifestyle behaviour and Douglas [11] described most GPs think health promotion is an important part of their job. However, not all studies have found positive attitudes among GPs. Jacobsen [22] concluded that GPs are frustrated due to the excessive expectations on the part of health policymakers. This was also a topic in our study, GPs said that they did not experience optimal support of the government either. Lawlor [8] stated that GPs are sceptical because they doubt their ability to be effective and mentioned that social, cultural and environmental factors are the most important determinants of population health. This vision corresponds with the vision of the GP type ‘ignorer’, one of six types identified in this pilot study.

Although our pilot study is small and explorative, we found a variation of topic areas. We categorized these different themes in types of GPs, but this categorisation is not stringent. Since this is a explorative pilot study, other types can be added to this categorisation as well. In the literature different types of GPs were also described. For instance Bucks [35] who identified five different clusters of GPs, varying from the GPs who is traditional in his/ her approach but at the same time speculative in taking risks, to the GP who is doctor centred in approach, but who is not prepared to take risks with patients health, preferring caution and prevention. Laws [23] describes four different roles of GPs (‘outside of professional role’, ‘the gatekeeper’, ‘the informer’ and ‘helper’) and how they influence the implementation of lifestyle risk factors. There are similarities with our findings, for instance the first role corresponds with the role ‘ignorer’ and the third one with ‘adviser’. However, in our study we found a wider range of different attitudes.

In this pilot study the topics brought up by PNs were more the same, which has also been found in some other studies. Steptoe [36] for example concluded that the majority of the GPs and PNs endorsed the statement that PNs are the most appropriate people to carry out health promotion.

This study has certain limitations. We used purposive sampling to find GPs and PNs, but we did not find GPs in solo practices who were willing to participate. Not only because the number of solo practices is declining, but also because of non-response and lack of time, we could not spend more time on finding solo practices. Therefore, only GPs in healthcare centres and general practices were interviewed.

Also, selection bias could have resulted from a non-response. It may be that GPs and PNs who participated in this study are more engaged in addressing lifestyle issues than participants who did not participate. The response rates among Dutch GPs in studies is generally low, probably because of the extension of their tasks, limited time to participate in studies and ‘research tiredness’ [37]. However, low response rates in GPs is not typically Dutch, Templeton, Creavin and Kaner also concluded it is becoming difficult to encourage GPs to participate in studies [38-40].

Due to the limited time of the GPs it was not only difficult to find general practices, but also to conduct in depth interviews of 60 minutes. Therefore we conducted interviews of 30 minutes. However, we did find a great variety in answers. No new themes were identified in the last three interviews so we assume that saturation was reached or almost reached. Yet, socially desirable answers or recall bias with respect to attitudes and barriers and facilitators is still possible.

### Implication for future research and clinical practice

In this study we highlighted 41 barriers mentioned by GPs and PNs with respect to health promoting activities in general practices. Future studies will be necessary to explore how (national) health promoting programs could be optimally implemented in general practices. Moreover, more studies are needed to assess the role of the GP umbrella organizations with respect to the development, dissemination and implementation of lifestyle interventions, because GPs and PNs stated they can advocate and spread proven effective health promotion programs in general practices on a continuous base. Furthermore, this was a small explorative pilot study. More in depth interviews are necessary to provide more insight in the different types of attitudes of GPs and PNs.

It is difficult to find solutions for the identified barriers in this study. Investing in collaboration between different disciplines in general practice and addressing the advocating role that umbrella GP organisations can have to promote lifestyle interventions could be strategies to increase health promotion activities. Next to this, a consistent national health policy to promote healthy behaviours is necessary. Furthermore, in this study enthusiasm of PNs to carry out lifestyle interventions was identified as a facilitator. The role and position of PNs should be further explored to gain the most benefit from them.

## Conclusion

GPs and PNs all say they discuss lifestyle issues with their patients. Referring patients to dieticians and physiotherapists was mentioned as an important activity to promote a healthy lifestyle. The number and the origin of health promotion activities that are organized and offered from within the general practice itself varies. Several barriers and facilitators with respect to health promotion were identified. The attitudes of GPs regarding health promotion were categorized as ignoring, advising, confirming, evangelising, interfering and nurturing. This explorative pilot study points out that, in general, GPs and PNs do carry out health promotion activities, but they experience barriers to fulfil this task.

## Competing interests

The author(s) declare that they have no competing interests.

## Authors’ contribution

Study design: WG, IvdG and TvA. Advice on the study design: TV. Data collection and analysis: WG and IvdG. Supervision TvA and TV. Manuscript preparation: WG; critical comments: IvdG, TvA and TV. All authors read and approved the final manuscript.

## Pre-publication history

The pre-publication history for this paper can be accessed here:

http://www.biomedcentral.com/1471-2296/14/20/prepub
